# Parental control and college students’ depressive symptoms: A latent class analysis

**DOI:** 10.1371/journal.pone.0287142

**Published:** 2024-02-07

**Authors:** Woosang Hwang, Eunjoo Jung, Narges Hadi, Seonghee Kim

**Affiliations:** 1 Department of Human Development and Family Sciences, Texas Tech University, Lubbock, TX, United States of America; 2 Department of Human Development and Family Science, Syracuse University, Syracuse, NY, United States of America; 3 Research Institute for Liberal Education, Yonsei University, Incheon, South Korea; Centre for Demographic Studies, SPAIN

## Abstract

We examined how maternal and paternal parental control (helicopter parenting, behavioral control, and psychological control) among college students are related to their depressive symptoms. We collected data from college students who attended a private university in Upstate New York (*n* = 455) and analyzed it using three-step latent class analysis. Latent class analysis identified four parental control latent classes: *weak parental control*, *strong psychological control*, *strong helicopter parenting*, and *strong parental control*. College students in the *weak parental control* class reported lower depressive symptoms than those in *strong psychological control* and *strong parental control* classes. In addition, college students in the *strong helicopter parenting* class reported lower depressive symptoms than those in *strong psychological control* and *strong parental control* classes. College students’ depressive symptoms were not significantly different between *weak parental control* and *strong helicopter parenting* classes. Future researchers are encouraged to continue to acknowledge the characteristics of different forms of parental control and their influence on college students’ quality of life in the transition to adulthood, considering the unique approaches of both parents.

## Introduction

The mental well-being of college students is critical to their academic and social success [1]. Although college students may live independently and away from their parents, parents continue to have a significant influence on their lives [2, 3]. Parenting practices have a direct and indirect impact on children’s quality of life, even after they have grown up [2–6]. The developmental systems theory highlights the importance of parental factors to family dynamics and their effect on an individual’s growth and development [7]. According to this theory, the family environment continues to affect college students’ functioning through complex interactions between parenting practices and family characteristics [7–10]. As parents continue to be involved in their emerging adult children’s lives, their parenting practices are likely to impact their children’s mental health [4, 5, 11, 12]. While some parents prioritize their children’s autonomy and self-exploration, others engage in controlling parenting, which may be inappropriate for their adult children [5]. Research has shown that appropriate parenting practices, such as supporting autonomy and independent decision-making, are linked to better academic performance and mental health among emerging adult children [6, 13–17]. Conversely, inappropriate parenting practices can result in negative outcomes, such as a sense of helplessness and dissatisfaction with life [11, 18–20]. A theoretical understanding of the influence of parenting practices on emerging adults illustrates that it is critical to investigate the impact of various parenting practices on the mental health and well-being of emerging adult children to better support their transition to adulthood.

In recent years, controlling parenting practices for emerging adults have been the subject of considerable research attention [4, 5, 21]. Researchers have identified three major forms of controlling parenting practices for emerging adult children: behavioral control, psychological control, and helicopter parenting [3, 5, 19]. Behavioral control involves parents’ monitoring, structuring, and directing children’s behaviors and daily activities. While this approach is commonly used for young children, it becomes unsuitable as children mature [22]. Behavioral control has been associated with negative outcomes in adolescents and emerging adults, such as low self-esteem, heightened self-criticism, and victimization by peers [23, 24]. In contrast, psychological control involves parents intruding on and manipulating children’s feelings, emotions, and thinking. Studies have found that psychological control is significantly associated with poor same-sex peer competence and romantic partner social competence, eating disorder symptoms, exercise dependence, and risk behaviors [25–27]. Helicopter parenting, which is characterized by overprotective and micromanaging behaviors, involves parents solving problems for their children and controlling their daily activities [3, 5, 11]. It’s important to note that not all forms of parental control have the same impact on child outcomes, and differences exist among them [28]. Helicopter parenting is considered qualitatively different from behavioral and psychological control because helicopter parents provide relatively high levels of support to their children. Therefore, it is related to but distinct from psychological and behavioral control in its impact on college students [3, 5].

Studies have indicated that emerging adults who experience helicopter parenting may face negative consequences, such as externalizing and internalizing problems, depression, impulsivity, and a decline in social competence [4, 5, 10, 19, 25, 26, 29]. Nevertheless, helicopter parenting may have fewer negative or neutral effects during the early stages of development and may become inappropriate as children grow and seek independence. The concern with helicopter parenting is that some parents continue to exhibit micromanaging and overprotective behavior even as their children reach developmental stages, like emerging adulthood, where they should be encouraged to gain autonomy [21].

The presence of depressive symptoms in college students is a crucial determinant of their academic and social functioning [1]. Previous studies have revealed that parental controlling behaviors are a significant contributor to the occurrence of depressive symptoms in emerging adults during their college years [30–32]. Although controlling parenting practices are generally considered unfavorable for emerging adult children, their impact on college students’ outcomes is not straightforward. Some studies have shown that helicopter parenting has negative effects on psychological well-being, family satisfaction, and academic performance, while others have found a positive association between helicopter parenting and psychological adjustment among college students who receive high levels of warmth and support from their parents [15, 16, 28, 33–36]. While both behavioral control and psychological control are considered risk factors for the development of emerging adults, there are positive aspects of helicopter parenting that may not have negative associations with children’s development outcomes [14]. For this reason, it is suggested parental controlling behaviors could be multidimensional [20].

Although there has been increasing interest in parental control over adult children, studies based on the multidimensional approach are limited. Most studies have investigated the relationships between controlling practices and young adult children’s developmental outcomes using a variable-centered approach (e.g., regression) [35, 37]. However, a person-centered approach (e.g., latent class analysis) allows for a more in-depth understanding of the multidimensionality of parental control practices. Recent research utilizing the person-centered approach has identified various typologies of parenting practices, such as warm helicopter parents, controlling helicopter parents, low-involved parents, average parenting, and high helicopter parenting [20].

Researchers also suggest that parental controlling behaviors would vary according to parents’ gender [19, 20]. Studies have shown variability in mother-child and father-child relationships, with mothers typically perceived as more authoritative than fathers [5, 38]. Due to their greater involvement in their children’s daily and social lives, mothers may engage in higher levels of controlling behavior with their emerging adult children than fathers do. However, some studies have found that mothers and fathers are similarly engaged in their emerging adult children’s academic and social activities, and the differences between mothers and fathers in some interaction domains are not substantial [35, 39, 40].

Despite the increasing attention to parental control over adult children, research on the interrelatedness of the three controlling practices—behavioral control, psychological control, and helicopter parenting—using a latent class approach to elucidate their underlying constructs has been lacking. Consequently, it is imperative to explore the connections between different types of maternal and paternal control and depressive symptoms of college students to comprehend better how diverse forms of parental control can affect depressive symptoms [41]. Therefore, the current study addresses this gap by formulating pertinent aims and conducting an in-depth investigation of this topic. We have the following aims:

1. To uncover latent classes of parental control that capture combinations of maternal and paternal helicopter parenting, behavioral control, and psychological control among college students.
2. To examine whether these parental control latent classes are associated with college students’ depressive symptoms.

## Methods

### Participants

We recruited college students from undergraduate classes (primarily social science or liberal arts) at a private institution in Upstate New York. After receiving approval from the Institutional Review Board in February 2017, we collected data from March 2017 to February 2018. The participants were recruited in their classrooms, and they signed a written informed consent form and completed a self-report questionnaire regarding their mothers and fathers. Students who completed the questionnaire received extra credit as compensation. The response rate was 88.72% (488 out of 550 students). We selected 450 out of 488 students who reported both mothers’ and fathers’ parental control items. In addition, given that the current study focused on college students in emerging adulthood (18–29 years) [42], we excluded five students who aged over 30 years.

Participants’ demographic information is presented in Table 1. The mean age of the participants was 19.87 years, and 69.7% were female. Regarding race, 64.3% were White, 10.6% were Black, and 9.6% were Asian. Most participants were living separately from their parents.

**Table 1:** Participants’ demographic information (*n* = 445).

Variables	*M (SD)*	*n* (%)
Age	19.87 (1.40)	
Gender		
• Male		130 (29.2)
• Female		310 (69.7)
Race		
• White		286 (64.3)
• Black		47 (10.6)
• Hispanic		27 (6.1)
• Asian		56 (9.6)
• Others		28 (6.2)
Parents’ Marital Status		
• Legally married		290 (65.2)
• Separated		16 (3.6)
• Divorced		65 (14.6)
• Never married		64 (14.4)
• Others		8 (1.8)
Family Structure		
• Biological mothers		403 (90.6)
• Stepmother		27 (6.1)
• Adoptive mother		7 (1.6)
• Biological father		376 (84.5)
• Stepfather		39 (8.8)
• Adoptive father		7 (1.6)
Current living arrangement		
• Living on campus (e.g., dormitory)		287 (64.5)
• Living off campus, not with parents		136 (30.6)
• Living off campus with parents		16 (3.6)
• Others		3 (0.7)
Parents’ annual household income		
• Under $20,000		17 (3.8)
• $20,001-$40,000		44 (9.9)
• $40,001-$60,000		32 (7.2)
• $60,001-$80,000		54 (12.1)
• $80,001-$100,000		70 (15.7)
• $100,001 or more		201 (45.2)

### Measures

#### Helicopter parenting

Maternal and Paternal helicopter parenting were separately measured using five items from Padilla-Walker and Nelson’s [28] Helicopter Parenting Scale. An example item was “My mother/father makes important decisions for me (e.g., where I live, where I work, what classes I take).” Response options ranged from 1 (*not at all*) to 5 (*a lot*). The Cronbach’s alpha of the five items was .79 for mothers and .78 for fathers.

#### Behavioral control

Maternal and paternal behavioral control were separately measured using five items from Padilla-Walker and Nelson’s [28] Behavioral Control Scale, which was adopted from Kerr and Stattin [43]. An example item was “My mother/father tries to limit or control who my friends are.” Response options ranged from 1 (*not at all*) to 5 (*a lot*). The Cronbach’s alpha for the five items was .82 for mothers and .79 for fathers.

#### Psychological control

Maternal and paternal psychological control were separately measured using four items from Padilla-Walker and Nelson’s [28] Psychological Control Scale, which was adopted from Morris et al. [44]. An example item was “My mother/father brings up past mistakes when she/he criticizes me.” Response options ranged from 1 (*not at all*) to 5 (*a lot*). The Cronbach’s alpha for the four items was .84 for mothers and .80 for fathers.

#### Depressive symptoms

Depression was measured using ten items from the Center for Epidemiologic Studies Depression Scale Short Form [45, 46]. An example item was “I felt depressed.” Response options ranged from 1 (*rarely or none of the time*) to 4 (*most or all of the time*). The Cronbach’s alpha for the 10 items was .80.

#### Control variables

Based on previous studies, we included children’s characteristics—age, gender (0 = *male*, 1 = *female*), and race (0 = *other racial groups*, 1 = *White*)—and parents’ characteristics—marital status (0 = *others*, 1 = *legally married*) and annual income,which are strongly associated with the parent-child relationship and college students’ mental health—as control variables [47–52]. Parents’ variables were separately measured for mothers and fathers. Considering that college students would not be familiar with their parents’ exact incomes, we used a categorical income question instead of an open-ended question to reduce missing values and measurement error [53]. The response options for parents’ annual income ranged from 1 (*under $20*,*000*) to 6 *($100*,*001 or more*).

### Data analysis

We conducted a latent class analysis using Latent Gold 6.0 to identify unobserved parental control latent classes. Latent class analysis “provides a framework for describing population heterogeneity in terms of differences across individuals on a set of behaviors or characteristics, as opposed to describing the variability of a single variable” [54, p.59]. We created six indicators for mean scores of maternal and paternal helicopter parenting (five items), behavioral control (five items), and psychological control (four items). Given that all indicators were ordinal variables, we initially conducted a latent profile analysis [54]. However, the optimal number of latent profiles was not identified. Regarding this issue, researchers suggested a dichotomization of ordinal indicators when they are skewed [55, 56]. We found that four out of six indicators were strongly skewed (see Table 2). Therefore, we dichotomized the six indicators as low [range 1(*not at all*)-2(*slightly*)] and high [range 2.1-5(*a lot*)) and conducted a latent class analysis. Regarding model selection, we used three information criteria: Bayesian Information Criterion (BIC), Consistent Akaike Information Criterion (CAIC), and entropy. We chose the optimal number of latent classes based on low BIC and CAIC values and entropy values over .8 [57].

**Table 2:** Descriptive results among study variables (*n* = 445).

Variables	*Range*	*M* (*SD*)	*n (%)*
Maternal helicopter parenting	1–5	2.34 (.86)	
• Low group			194 (43.6)
• High group			244 (54.8)
Maternal behavioral control	1–5	1.77 (.80)^a^	
• Low group			333 (74.8)
• High group			105 (23.6)
Maternal psychological control	1–5	1.89 (.97)^a^	
• Low group			301 (67.6)
• High group			140 (31.5)
Paternal helicopter parenting	1–5	2.03 (.82)	
• Low group			259 (58.2)
• High group			179 (40.2)
Paternal behavioral control	1–5	1.64 (.70)^a^	
• Low group			350 (78.7)
• High group			86 (19.3)
Paternal psychological control	1–5	1.73 (.86)^a^	
• Low group			331 (74.4)
• High group			111 (24.9)
Depressive symptoms	1–4	1.99 (.55)	

*Note*. a = strongly skewed.

After identifying the optimal number of latent classes, we conducted a Bolck-Croon-Hagenaars (BCH) [58] three-step analysis using Latent Gold 6.0 to examine whether latent classes are associated with college students’ depressive symptoms. The BCH approach helps adjust classification errors while examining the association between latent class membership and outcome variables [58]. In this step, we modeled depressive symptoms (mean score of ten items) as a function of class membership weighted by latent class membership probabilities and controlling for college students’ and their parents’ demographic characteristics (age, gender, race, parents’ marital status, and parents’ annual income). Lastly, we used full information maximum likelihood (FIML) approach to address missing values.

## Results

### Descriptive analysis

The results of descriptive analysis are presented in Table 2. The mean scores of six parental control variables were lower than the mid-point (3). In addition, the mean score of depressive symptoms was lower than the mid-point (2.5).

### Latent class analysis

**Fig 1:**
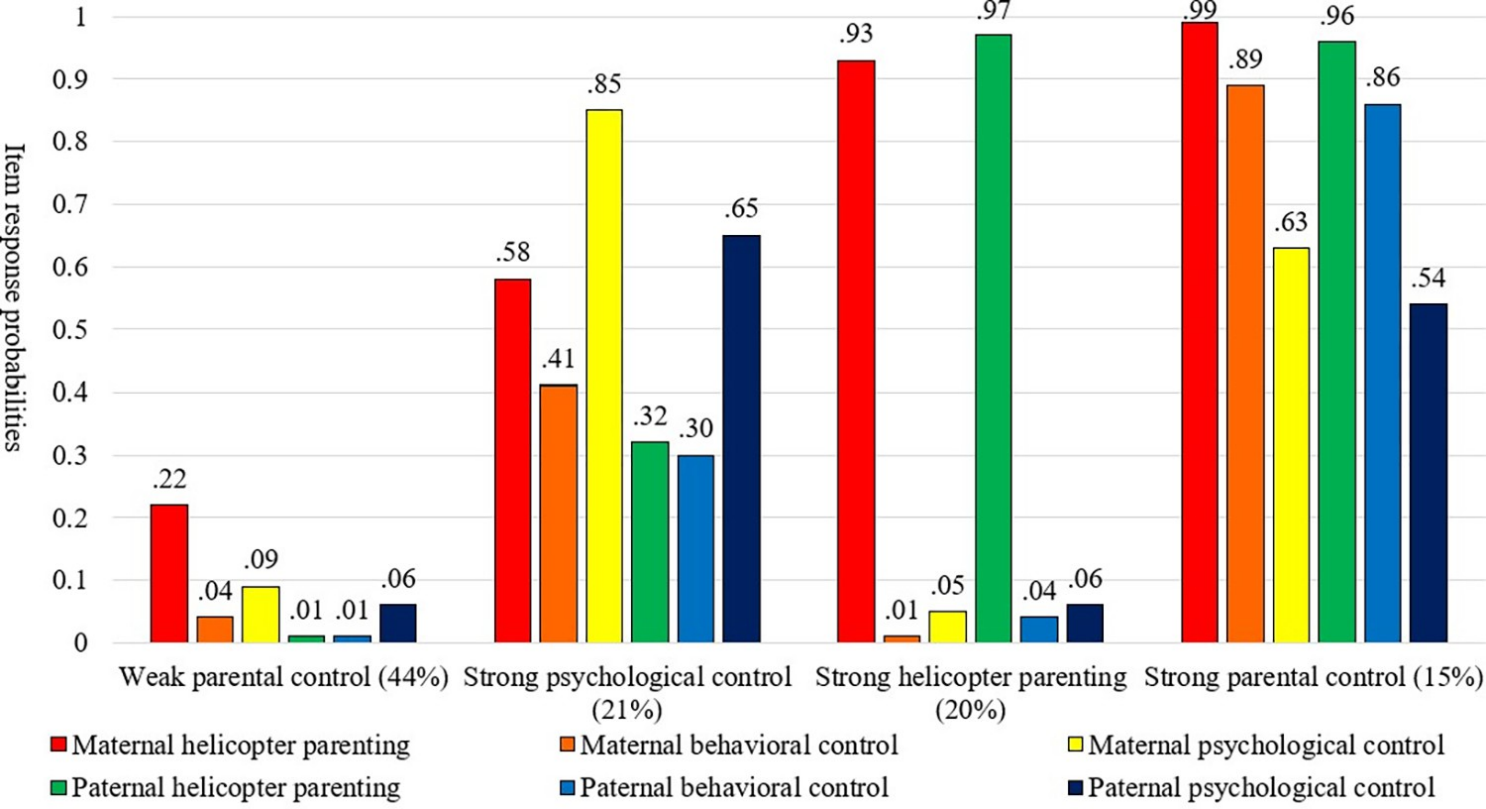
Item response and latent class probabilities.

The results of latent class analysis are presented in Table 3. Based on three information criteria, we selected a four-class model as the best-fitting model. Item response and latent class probabilities are visualized in Fig 1. Using .5 cut-off criteria, we labeled four classes as follows. The first class was *weak parental control* (44%). In this class, item response probabilities of all maternal and paternal control indicators were under .5. The second class was *strong psychological control* (21%). In this class, item response probabilities of maternal and paternal psychological control indicators were over .5. The third class was *strong helicopter parenting* (20%). In this class, item response probabilities of maternal and paternal helicopter parenting were over .5. The fourth class was *strong parental control* (15%). In this class, all maternal and paternal control indicators were over .5.

**Table 3:** Latent class analysis statistics and fit indices.

	Classes (n)	BIC	AIC	Entropy
Model 1	1	3195.43	3201.43	-
Model 2	2	2839.39	2852.39	.77
Model 3	3	2742.34	2762.34	.82
Model 4	4	**2732.26**	**2759.26**	.82
Model 5	5	2743.17	2777.17	.82
Model 6	6	2758.99	2799.99	.85

*Note*. Bolded values indicate the best fit for each respective statistic. BIC = Bayesian Information Criterion. CAIC = Consistent Akaike Information Criterion.

### Association between latent classes and depressive symptoms

The results of multivariate regression are presented in Table 4 (reference group: weak parental control). We found that college students in the *strong psychological control* and *strong parental control* latent classes reported higher depressive symptoms than those in the *weak parental control* latent class (*p* < .001; *p* < .05). However, college students’ depressive symptoms were not significantly different between the *strong helicopter parenting* and *weak parental control* classes.

**Table 4:** Associations between latent classes and depressive symptoms.

	Depressive symptoms
**Variable**	*b* (*se*)
Latent classes (ref: weak parental control)	
• Strong psychological control	.30 (.08)***
• Strong helicopter parenting	-.01 (.07)
• Strong parental control	.18 (.09)*
Control variables	
• Age	.01 (.01)
• Female (vs. male)	-.03 (.06)
• White (vs. others)	-.04 (.06)
• Married parents (vs. others)	-.01 (.05)
• Parents’ income	-.01 (.01)

*Note*: *p < .05.

**p < .01.

***p < .001.

We additionally tested paired comparisons to investigate other contrasts in latent classes that were not captured by the multivariate regression (see Table 5). We found that college students in the *strong helicopter parenting* latent class reported lower depressive symptoms than those in the *strong psychological control* and *strong parental control* latent classes (*p* < .01; *p* = .055). College students’ depressive symptoms were not significantly different between the *strong psychological control* and *strong parental control* latent classes.

**Table 5:** Results of paired comparison test of depressive symptoms across latent classes.

	Class 1 Weak parental control	Class 2 Strong psychological control	Class 3 Strong helicopter parenting	Class 4 Strong parental control	
	*M* (*SE*)	*M* (*SD*)	*M* (*SD*)	*M* (*SD*)	*p-value*
Depressive symptoms	1.90 (.04)	2.20 (.06)	1.89 (.06)	2.09 (.08)	2 > 1***
					2 > 3**
					4 > 1*
					4 > 3^†^

*Note*: Covariates were controlled but not presented in Table. ^†^*p* = .055.

*p < .05.

**p < .01.

***p < .001.

## Discussion

Parenting of college students has received less attention in the field of family studies than parenting of children or adolescents, despite its importance. Family environment and parenting practices are as important for college students as for any other developmental age level, because college students’ life outcomes are substantially influenced by the interaction between family environment and individual characteristics [5, 11]. This study was designed based on the relational developmental systems perspective [7, 8] to identify uncovered subgroups of parental control latent classes based on six indicators (maternal and paternal helicopter parenting, behavioral control, and psychological control) among college students, and to examine whether these parental control latent classes are associated with college students’ depressive symptoms. In doing so, we were able to better understand the importance of parental control in college students’ psychological well-being within the familial context.

The first aim of this study was to uncover latent classes of parental control that captured combinations of maternal and paternal helicopter parenting, behavioral control, and psychological control among college students. We identified four parental control latent classes. The *strong helicopter parenting* and *strong psychological control* classes represent distinct parental control patterns compared to the *strong parental control* and *weak parental control* classes. Interestingly, maternal and paternal parental control shows similar patterns in the four latent classes. For example, in the *strong helicopter parenting* class, when mothers’ helicopter parenting was high, fathers’ helicopter parenting was also high. Given that mothers’ and fathers’ parenting styles are interdependent [59], it can be interpreted that one parent’s controlling parenting would affect the other’s controlling parenting.

The second aim was to examine whether these parental control latent classes are associated with college students’ depressive symptoms. We found that college students in the *weak parental control* class reported lower depressive symptoms compared to those in the *strong psychological control* and *strong parental control* classes. However, college students’ depressive symptoms were not significantly different between the *weak parental control* and *strong helicopter parenting* classes. Furthermore, college students in the *strong helicopter parenting* class reported lower depressive symptoms compared to those in *strong psychological control* and *strong parental control* classes. These results are linked to the perspective of relational developmental systems theory in that college students’ mental health is influenced by parental practices [8]. Given that college students are still financially and emotionally dependent on their parents [3, 5, 60] and that they are still significantly influenced by their interactions with their parents and family environments, our findings show that parental control serves as an important component of their quality of life.

In addition, our finding supports Padilla-Walker and Nelson’s [28] finding that helicopter parenting is distinguished from behavioral and psychological control. The similarity between the *strong helicopter parenting* and *weak parental control* classes is low levels of maternal and paternal behavioral and psychological control. It is possible that high levels of helicopter parenting but low levels of behavioral and psychological control would be less harmful to college students’ depressive symptoms than high levels of behavioral and psychological control. In addition, college students in the *strong psychological control* class reported higher depressive symptoms than those in weak parental control and strong helicopter parenting classes. This result is consistent with previous studies showing that psychological control is more harmful to young adult children’s mental health than other forms of parental control [5, 61].

### Limitations

Several limitations of the study should be considered. First, we recruited participants from a private university in Upstate New York. As a result, participants’ parents usually had high socioeconomic status, and the findings of this study may not generalize to college students in public universities, community colleges, or other types of higher educational institutions. Second, the majority of participants were white females. Therefore, it is possible that the constructs of this study—helicopter parenting, behavioral control, and psychological control—may lack ecological validity. Third, although we controlled for parents’ marital status in the analysis; this method is insufficient to explain the effects of diverse family structures, such as step-families and adoptive families. Fourth, given that information about the three forms of parental control and the parent-child relationship relied on children’s self-reports, the possibility that there could be a discrepancy between parents’ and children’s perceptions regarding parental control should be considered. Fifth, our results should be carefully interpreted because this study was cross-sectional. We recommend that future studies address whether parental control is related to college students’ depressive symptoms using longitudinal and dyadic data.

### Implications

Despite these limitations, our findings highlight an important practical implication for parenting college students, particularly for improving emerging adult children’s mental health. We examined three forms of parental control: helicopter parenting, behavioral control, and psychological control. We found that helicopter parenting is a distinct construct from behavioral and psychological control. We speculate that helicopter parenting includes both control and support for emerging adult children, whereas psychological and behavioral control may not. From the parents’ perspective, they may be willing to give educational resources and life advice to their children to facilitate academic and occupational success; this parental involvement and social support may be a critical component of helicopter parenting. From the emerging adult children’s perspective, parental involvement and support are helpful for their academic achievement and job searches. With this foundation, researchers and family practitioners are encouraged to examine the impact of different forms of parental control on college students’ other developmental outcomes.

At the same time, emerging adult children’s ability to handle their mothers’ and fathers’ controlling parenting across different cultural contexts should be carefully considered. Parenting may be viewed differently by people with different socio-cultural backgrounds. For example, helicopter parenting is construed in a positive manner in Chinese culture [62]. Differences between families in different socio-cultural contexts will need to be more carefully considered when interpreting these results. In addition, it is important to consider that results may be different depending on parents’ different family roles in different cultural contexts, as these interactions may result in different outcomes [7, 63]. Therefore, more cross-cultural studies are required to examine the effect of helicopter parenting on college students’ well-being.

## Conclusion

In conclusion, different forms of parental control in emerging adulthood are uniquely related to college students’ mental health and can be largely explained by individual and familial contexts. Our findings uniquely contribute to the body of work on the nature and effects of parental control and suggest that parental control still has important implications for college students’ quality of life. In addition, our findings suggest that helicopter parenting, which is distinct from behavioral and psychological control, can be differently associated with college students’ mental health compared to other types of parental control. Family practitioners and educators are encouraged to acknowledge the complex nature of helicopter parenting and to develop appropriate parenting styles for young adults’ successful transition to adulthood.

## References

[1] Oswalt SB, Lederer AM, Chestnut-Steich K, Day C, Halbritter A, Ortiz D. Trends in college students’ mental health diagnoses and utilization of services, 2009–2015. J Am Coll Health. 2020;68: 41–51. doi: 10.1080/07448481.2018.1515748 30355071

[2] Kouros CD, Pruitt MM, Ekas NV, Kiriaki R, Sunderland M. Helicopter parenting, autonomy support, and college students’ mental health and well-being: The moderating role of sex and ethnicity. J Child Fam Stud. 2017;26: 939–949. doi: 10.1007/s10826-016-0614-3 31832009 PMC6907082

[3] Nelson LJ, Padilla-Walker LM, Son D. Helicopter parenting, parental control, and moral development during emerging adulthood. In: Laible D, Padilla-Walker LM, Carlo &. G, editors. Oxford Handbook of Parenting and Moral Development. NY: Oxford University Press; 2019. pp. 355–74.

[4] Cui M, Hong P, Jiao C. Overparenting and emerging adult development: A systematic review. Emerg Adulthood. 2022;10: 1076–1094. doi: 10.1177/21676968221108828

[5] Padilla-Walker LM, Nelson LJ. Parenting emerging adults. In: Bornstein M, editor. Handbook of Parenting. NJ: Lawrence Erlbaum; 2019. pp. 168–90.

[6] Reed K, Duncan JM, Lucier-Greer M, Fixelle C, Ferraro AJ. Helicopter parenting and emerging adult self-efficacy: Implications for mental and physical health. J Child Fam Stud. 2016;25: 3136–3149. doi: 10.1007/s10826-016-0466-x

[7] Lerner RM. Promoting positive human development and social justice: Integrating theory, research and application in contemporary developmental science. Int J Psychol. 2015;50: 165–173. doi: 10.1002/ijop.12162 25782450

[8] Overton WF. A new paradigm for developmental science: Relationism and relational-developmental systems. Appl Dev Sci. 2013;17: 94–107. doi: 10.1080/10888691.2013.778717 23834001

[9] Overton WF, Müller U. Development across the life span: Philosophy, concepts, theory. Lerner RM, Easterbrooks MA, Mistry &. J, editors. Handbook of psychology. 2012; 6:19–58.

[10] Reed K, Ferraro AJ, Lucier-Greer M, Barber C. Adverse family influences on emerging adult depressive symptoms: A stress process approach to identifying intervention points. J Child Fam Stud. 2015;24: 2710–2720. doi: 10.1007/s10826-014-0073-7

[11] Lindell AK, Campione-Barr N, Killoren SE. Implications of parent–child relationships for emerging adults’ subjective feelings about adulthood. J Fam Psychol. 2017;31: 810–820. doi: 10.1037/fam0000328 28517943

[12] Turner LA, Faulk RD, Garner T. Helicopter parenting, authenticity, and depressive symptoms: A mediation model. J Genet Psychol. 2020;181: 500–505. doi: 10.1080/00221325.2020.1775170 32552440

[13] Darlow V, Norvilitis JM, Schuetze P. The relationship between helicopter parenting and adjustment to college. J Child Fam Stud. 2017;26: 2291–2298. doi: 10.1007/s10826-017-0751-3

[14] Inguglia C, Ingoglia S, Liga F, Coco AL, Cricchio MGL, Musso P, et al. Parenting dimensions and internalizing difficulties in Italian and US emerging adults: The intervening role of autonomy and relatedness. J Child Fam Stud. 2016;25: 419–431. doi: 10.1007/s10826-015-0228-1

[15] Segrin C, Woszidlo A, Givertz M, Bauer A, Taylor Murphy M. The association between overparenting, parent-child communication, and entitlement and adaptive traits in adult children. Fam Relat. 2012;61: 237–252. doi: 10.1111/j.1741-3729.2011.00689.x

[16] Jung E, Hwang W, Kim S, Sin H, Zhao Z, Zhang Y, et al. Helicopter parenting, autonomy support, and student wellbeing in the United States and South Korea. Journal of Child and Family Studies. 2020 Feb;29:358–73. doi: 10.1007/s10826-019-01601-7

[17] Pedersen DE. Parental autonomy support and college student academic outcomes. J Child Fam Stud. 2017;26: 2589–2601. doi: 10.1007/s10826-017-0750-4

[18] Luebbe AM, Mancini KJ, Kiel EJ, Spangler BR, Semlak JL, Fussner LM. Dimensionality of helicopter parenting and relations to emotional, decision-making, and academic functioning in emerging adults. Assessment. 2018;25: 841–857. doi: 10.1177/1073191116665907 27561986

[19] Nelson LJ, Padilla-Walker LM, Christensen KJ, Evans CA, Carroll JS. Parenting in emerging adulthood: an examination of parenting clusters and correlates. J Youth Adolesc. 2011;40: 730–743. doi: 10.1007/s10964-010-9584-8 20809102

[20] Padilla-Walker LM, Son D, Nelson LJ. Profiles of helicopter parenting, parental warmth, and psychological control during emerging adulthood. Emerg Adulthood. 2021;9: 132–144. doi: 10.1177/2167696818823626

[21] Zhang Y, Hwang W, Jung E, Kim SE, Sin HL. Helicopter parenting, parental psychological and behavioral control revisited: Assessing constructs across the United States and South Korea. J Comp Fam Stud. 2020;51: 59–83. doi: 10.3138/jcfs.51.1.004

[22] Bacikova-Sleskova M, Benka J, Orosova O. Parental behavioral control and knowledge in early adolescence. A person-oriented approach. Curr Psychol. 2019;38: 1–10. doi: 10.1007/s12144-019-00214-z

[23] Gittins CB, Hunt C. Parental behavioral control in adolescence: How does it affect self-esteem and self-criticism? J Adolesc. 2019;73: 26–35. doi: 10.1016/j.adolescence.2019.04.006 30953842

[24] Li D, Zhang W, Wang Y. Parental behavioral control, psychological control and Chinese adolescents’ peer victimization: The mediating role of self-control. J Child Fam Stud. 2015;24: 628–637. doi: 10.1007/s10826-013-9873-4

[25] Moilanen KL, Manuel ML. Parenting, self-regulation and social competence with peers and romantic partners. J Appl Dev Psychol. 2017;49: 46–54. doi: 10.1016/j.appdev.2017.02.003

[26] Urry SA, Nelson LJ, Padilla-Walker LM. Mother knows best: Psychological control, child disclosure, and maternal knowledge in emerging adulthood. J Fam Stud. 2011;17: 157–173. doi: 10.5172/jfs.2011.17.2.157

[27] Costa S, Hausenblas HA, Oliva P, Cuzzocrea F, Larcan R. Maladaptive perfectionism as mediator among psychological control, eating disorders, and exercise dependence symptoms in habitual exerciser. J Behav Addict. 2016;5: 77–89. doi: 10.1556/2006.5.2016.004 28092194 PMC5323000

[28] Padilla-Walker LM, Nelson LJ. Black hawk down? Establishing helicopter parenting as a distinct construct from other forms of parental control during emerging adulthood. J Adolesc. 2012;35: 1177–1190. doi: 10.1016/j.adolescence.2012.03.007 22503075

[29] Manzeske DP, Stright AD. Parenting styles and emotion regulation: The role of behavioral and psychological control during young adulthood. J Adult Dev. 2009;16: 223–229. doi: 10.1007/s10804-009-9068-9

[30] Dong XX, Liang G, Li DL, et al. Association between parental control and depressive symptoms among college freshmen in China. The chain mediating role of chronotype and sleep quality. J Affect Disord. 2022;317: 256–264. doi: 10.1016/j.jad.2022.08.091 36055527

[31] Feng B, Zhang Y, Zhang L, Xie X, Geng W. Change in the level of depression among Chinese college students from 2000 to 2017: A cross-temporal meta-analysis. Soc Behav Personal. 2020;48: 1–16. doi: 10.2224/sbp.8832

[32] Wang J, Lai R, Yang A, Yang M, Guo Y. Helicopter parenting and depressive level among non-clinical Chinese college students: A moderated mediation model. J Affect Disord. 2021;295: 522–529. doi: 10.1016/j.jad.2021.08.078 34509067

[33] Hong P, Cui M. Helicopter parenting and college students’ psychological maladjustment: The role of self-control and living arrangement. J Child Fam Stud. 2020;29: 338–347. doi: 10.1007/s10826-019-01541-2

[34] Schiffrin HH, Erchull MJ, Sendrick E, Yost JC, Power V, Saldanha ER. The effects of maternal and paternal helicopter parenting on the self-determination and well-being of emerging adults. J Child Fam Stud. 2019;28: 3346–3359. doi: 10.1007/s10826-019-01513-6

[35] Schiffrin HH, Liss M. The effects of helicopter parenting on academic motivation. J Child Fam Stud. 2017;26: 1472–1480. doi: 10.1007/s10826-017-0658-

[36] Fingerman KL, Cheng Y-P, Wesselmann ED, Zarit S, Furstenberg F, Birditt KS. Helicopter parents and landing pad kids: Intense parental support of grown children. J Marriage Fam. 2012;74: 880–896. doi: 10.1111/j.1741-3737.2012.00987.x 26336323 PMC4553417

[37] Barber BK, Xia M, Olsen JA, McNeely CA, Bose K. Feeling disrespected by parents: Refining the measurement and understanding of psychological control. J Adolesc. 2012;35: 273–287. doi: 10.1016/j.adolescence.2011.10.010 22177194

[38] Conrade G, Ho R. Differential parenting styles for fathers and mothers: Differential treatment for sons and daughters. Aust J Psychol [Internet]. 2001;53: 29–35. doi: 10.1080/00049530108255119

[39] Cui M, Janhonen-Abruquah H, Darling CA, Carlos Chavez FL, Palojoki P. Helicopter parenting and young adults’ well-being: A comparison between the United States and Finland. Cross Cult Res. 2019;53: 410–427. doi: 10.1177/1069397118802253

[40] Lamb ME, Lewis C. Father-child relationships. In: Cabrera N, Tamis- Lemonda CS, editors. Handbook of father involvement. New York: Psychology Press; 2013. pp. 119–34.

[41] Hwang W, Jung E. Helicopter parenting versus autonomy supportive parenting? A latent class analysis of parenting among emerging adults and their psychological and relational well-being. Emerging Adulthood. 2022 Jun;10(3):731–43. doi: 10.1177/2167696821100049

[42] Arnett JJ. Emerging adulthood: The winding road from the late teens through the twenties. Cary, NC: Oxford University Press; 2014.

[43] Kerr M, Stattin H. What parents know, how they know it, and several forms of adolescent adjustment: further support for a reinterpretation of monitoring. Dev Psychol. 2000;36: 366–380. doi: 10.1037/0012-1649.36.3.366 10830980

[44] Morris AS, Steinberg L, Sessa FM, Avenevoli S, Silk JS, Essex MJ. Measuring children’s perceptions of psychological control: developmental and conceptual considerations. In Barber BK, editor, Psychological control of children and adolescents, 125–159. American Psychological Association, 2002.

[45] Björgvinsson T, Kertz SJ, Bigda-Peyton JS, McCoy KL, Aderka IM. Psychometric properties of the CES-D-10 in a psychiatric sample. Assessment. 2013;20: 429–436. doi: 10.1177/1073191113481998 23513010

[46] Radloff LS. The CES-D scale a self-report depression scale for research in the general population. Applied Psychological Measurement. 1977;1: 385–401

[47] Amato PR, Dorius C. Fathers, children, and divorce. In: Lamb ME, editor. The role of the father in child development. Hoboken NJ: Wiley; 2010. pp. 177–200.

[48] Hwang W., & Jung E. (2021). Parenting practices, parent–child relationship, and perceived academic control in college students. Journal of Adult Development, 28, 37–49. doi: 10.1007/s10804-020-09346-0

[49] Crittenden PM. A dynamic-maturational model of attachment. Aust N Z J Fam Ther. 2006;27: 105–115. doi: 10.1002/j.1467-8438.2006.tb00704.x

[50] Langford W, Lewis C, Solomon Y, Warin J. Family understandings: Closeness, authority and independence in families with teenagers. In: Family Policy Centre & Joseph Rowntree Foundation. London; 2001.

[51] Ruble DN, Martin CL. Handbook of child psychology: Social, emotional, and personality development. Damon W, Eisenberg N, editors. John Wiley & Sons Inc; 1998.

[52] Silverstein M, Bengtson VL. Intergenerational solidarity and the structure of adult child‐parent relationships in American families. Am J Social. 1997;103: 429–460. doi: 10.1086/231213

[53] Lavrakas PJ. Encyclopedia of survey research methods. Sage Publications; 2008.

[54] Lanza ST, Cooper BR. Latent class analysis for developmental research. Child Dev Perspect. 2016;10: 59–64. doi: 10.1111/cdep.12163 31844424 PMC6914261

[55] Vasilenko SA, Espinosa‐Hernández G. Multidimensional profiles of religiosity among adolescents: Associations with sexual behaviors and romantic relationships. J Res Adolesc. 2019;29: 414–428. doi: 10.1111/jora.12444 31206883 PMC6581207

[56] Vasilenko SA. More than the sum of their parts: A dyad-centered approach to understanding adolescent sexual behavior. Sex Res Social Policy. 2022;19: 105–118. doi: 10.1007/s13178-020-00528-9 35990880 PMC9390880

[57] Nylund-Gibson K, Choi AY. Ten frequently asked questions about latent class analysis. Transl Issues Psychol Sci. 2018;4: 440–461. doi: 10.1037/tps0000176

[58] Bakk Z, Tekle FB, Vermunt JK. Estimating the association between latent class membership and external variables using bias-adjusted three-step approaches. Sociol Methodol. 2013;43: 272–311. doi: 10.1177/0081175012470644

[59] Tavassolie T, Dudding S, Madigan AL, Thorvardarson E, Winsler A. Differences in perceived parenting style between mothers and fathers: Implications for child outcomes and marital conflict. J Child Fam Stud. 2016;25: 2055–2068. doi: 10.1007/s10826-016-0376-y

[60] Hwang W, Kim I. Parental financial support and filial responsibility in emerging adulthood: A comparative study between the United States and South Korea. Journal of Youth Studies. 2016 Nov 25;19(10):1401–18. 10.1080/13676261.2016.1171833

[61] Bean RA, Barber BK, Crane DR. Parental support, behavioral control, and psychological control among African American youth: The relationships to academic grades, delinquency, and depression. J Fam Issues. 2006;27: 1335–1355. doi: 10.1177/0192513X06289649

[62] Leung JTY, Shek DTL. Unbroken homes: Parenting style and adolescent positive development in Chinese single-mother families experiencing economic disadvantage. Child Indic Res. 2018;11: 441–457. doi: 10.1007/s12187-016-9437-4

[63] Lamb ME. Fathers and child development: An introductory overview and guide. In: Lamb ME, editor. The role of the father in child development. NY: Wiley; 1997. pp. 1–18.

